# Human Neural Cells Transiently Express Reelin during Olfactory Placode Development

**DOI:** 10.1371/journal.pone.0135710

**Published:** 2015-08-13

**Authors:** M. Cristina Antal, Brigitte Samama, M. Said Ghandour, Nelly Boehm

**Affiliations:** 1 Institut d'Histologie, Faculté de Médecine, Université de Strasbourg, Strasbourg, France; 2 Fédération de Médecine Translationnelle de Strasbourg, Strasbourg, France; 3 Hôpitaux Universitaires de Strasbourg, Strasbourg, France; 4 Laboratoire d’Imagerie et de Neurosciences Cognitives, CNRS, UMR 7237, Strasbourg, France; 5 CNRS UMR 7357, Strasbourg, France; Duke University, UNITED STATES

## Abstract

Reelin, an extracellular glycoprotein is essential for migration and correct positioning of neurons during development. Since the olfactory system is known as a source of various migrating neuronal cells, we studied Reelin expression in the two chemosensory olfactory systems, main and accessory, during early developmental stages of human foetuses/embryos from Carnegie Stage (CS) 15 to gestational week (GW) 14. From CS 15 to CS 18, but not at later stages, a transient expression of Reelin was detected first in the presumptive olfactory and then in the presumptive vomeronasal epithelium. During the same period, Reelin-positive cells detach from the olfactory/vomeronasal epithelium and migrate through the mesenchyme beneath the telencephalon. Dab 1, an adaptor protein of the Reelin pathway, was simultaneously expressed in the migratory mass from CS16 to CS17 and, at later stages, in the presumptive olfactory ensheathing cells. Possible involvements of Reelin and Dab 1 in the peripheral migrating stream are discussed.

## Introduction

During development of the olfactory system, peripheral neurosensory axons arising from the invaginated placode reach the presumptive olfactory area of the telencephalon, thus mediating olfactory bulb (OB) development. In the main olfactory system, neurosensory cells in the olfactory epithelium (OE), sending axons to the main OB, make synapses with the dendrite of mitral/tufted cells, whereas in the accessory olfactory system, axons arising from the neurosensorial cells of the vomeronasal epithelium (VNE), lying in the vomeronasal organ (VNO), make synapses with mitral/tufted cells in the accessory olfactory bulb. However, in human, the VNO is present during embryonic/foetal development, contains neurosensory-like cells but is no more functional at birth [1].

The olfactory placode (OP), and later the VNO and OE are well known as sources of various migrating neurons toward the telencephalon; among them are GnRH neurons that migrate along the vomeronasal-terminal nerve (VN/TN) [2]. Several guidance molecules have been proposed for guiding olfactory axons and neurons through the mesenchyme toward the presumptive OB area and the septal region, respectively. Molecules involved in cell adhesion (anosmin1, laminin, heparane sulphate proteoglycan), transcription factors and neurotransmitters have been described in the literature (review in [3]).

Reelin has emerged as an extracellular glycoprotein essential for migration and correct positioning of cortical neurons and for establishment of correct neuronal circuitry during nervous system development; Reelin is expressed during development by different classes of neurons and especially by Cajal-Retzius cells in cortical layer I [4, 5]. During development, Reelin acts mainly through its VLDL and Apo2E receptors, initiating a signalling cascade where an adapter protein Dab 1 is phosphorylated and binds to the intracellular part of the receptor [6–9]. Reelin expression has also been detected in many other regions of the developing central nervous system in addition to cortical layer I, as well as in peripheral organs such as liver, lymphatic endothelial cells, pituitary pars intermedia, adrenal chromaffin cells [10, 11] and in the peripheral part of the olfactory system in mouse embryos [12,13].

Most data about Reelin pathway during development were obtained in rodents and comparisons with human development showed similarities but also some differences [14]. Since, at our knowledge, no data were available about the expression of the Reelin pathway components in the human olfactory system during the period of cell migration, we studied the expression of Reelin and Dab 1 at some stages of the embryonic/early foetal period in human. Here we show that some early neural cells transiently express Reelin at the time they leave the presumptive OE/VNE and that Dab 1 is present in the migratory cell mass and in the presumptive ensheathing cells in the absence of Reelin expression.

## Materials and Methods

### Biological samples

Twelve human embryos/foetuses ranging from Carnegie stage (CS) 15 to gestational weeks (GW) 12 from legal abortions and one normal brain and nasal medial wall (14 GW) from a foetus without macroscopic malformations addressed to the Pathology Service of the University Hospital for examination following abortion were studied. These embryos/foetuses were collected following requirements and regulations approved by the Medical Ethics Committee of the Faculty of Medicine of Strasbourg. Written informed maternal consents were obtained from an independent physician according to the procedure approved by the ethics committee. 1 CS15, 1 CS16, 1 CS17, 1 CS18, 2 CS 20–21, 3 CS23-24, 2 GW8, 1 GW12 and 1 GW14 embryos/foetuses were used. For the CS18 embryo, only nasal cavities could be studied. Developmental stage of embryos/foetuses was ascertained based on external morphology, peripheral organs and brain development according to O’Rahilly and Müller [15], Wolff-Quenot and Sick [16] and Feess-Higgins and Larroche [17]. Tissues were fixed in Bouin-Holland fixative (13 embryos/foetuses) or buffered formalin (1 foetus) and 5 μm paraffin sections were cut. Microscopic examination following haematoxylin-eosin (H.E.) staining showed no abnormalities and adjacent sections were further processed for immunocytochemistry and *in situ* hybridization (one formalin fixed GW8 embryo).

### Immunohistochemistry

Antibodies used were mouse monoclonal antibodies against beta-III tubulin (1/1000, Chemicon, Abcys, Paris, France), ICBP90 (1/2000, Proteogenix, Illkirch, France), Reelin-142 (1/1000), a generous gift from Pr. A. Goffinet [18] and rabbit polyclonal antibodies against calretinin (1/10000, Swant, Bellinzona, Switzerland), NSE (1/1000, Dakocytomation, Trappes, France), GnRH (two antibodies: Sanbio, Tebu, Le Perray en Yvelines, France, 1/10000 and LR1, 1/10000, a generous gift from Pr. R. Benoit, Montreal), PGP 9.5 (1/2000, Affinity Reagents, Fisher Scientific, Illkirch, France), Dab 1 and AC3 (1/1000, Santacruz Biotechnology, Tebu, Le Perray en Yvelines, France), S100 (1/1000 Dakocytomation, Trappes, France).

Microwave antigen retrieval (10 mM citrate buffer, pH 6.0) of paraffin sections was followed by incubation in blocking buffer; endogenous peroxydases were blocked (3% H2O2 for 10 min) and primary antibody incubated overnight at 4°C; a secondary biotinylated antibody incubation for 2 hours was followed by avidin-biotin complex incubation (Vectastain Elite kit, Vector Laboratories, Abcys, Paris, France). The peroxydase reaction product was revealed by VIP (Vector Laboratories, Abcys, Paris, France) as the chromogen. Sections were counterstained with methyl green, air-dried and cover slipped with Eukitt (Labonord, Templemars, France). Negative controls consisted in omission of the primary antibody, resulting in no staining.

For double immunostainings, sections were incubated simultaneously in anti-Reelin-142 (1/200) and anti-PGP9.5 (1/500), anti-calretinin (1/500), anti-GnRH (1/1000) or anti-Dab 1 (1/50) and the reaction was revealed by incubation in secondary either anti-mouse Alexa Fluor 568-labeled and anti-rabbit Alexa Fluor 488-labeled or anti-rabbit Alexa Fluor 568-labeled and anti-mouse Alexa Fluor 488-labeled antibodies (1/200, Molecular probes, Invitrogen, Cergy Pontoise, France). DAPI in mounting medium (Vectashield, Vector Laboratories, Abcys, Paris, France) was used for nuclei staining.

### Reelin *in situ* hybridization

#### RNA isolation and probe preparation

Total RNA and probe preparations by RT-PCR Dig labelling were performed as previously described [19]. Briefly, total RNA was isolated from C57BL/6 mouse brains using TRIzol reagent according to manufacturer instructions (GIBCO BRL, Cergy Pontoise, France). To eliminate contamination with genomic DNA, isolated RNAs were treated with DNaseI, RNase free (Amersham Pharmacia Biotech, Orsay, France). Before reverse transcribing the mRNA, we checked for genomic contamination by PCR using primers for genomic Reelin. cDNA was prepared from the total mouse brain RNA using Qiagen Omniscript kit (Qiagen, Hilden, Germany). Total RNA (1–2μg/reaction) in the presence of Oligo dT15 primers (Promega, Charbonnières-les Bains, France), and Omniscript Reverse Transcriptase was incubated at 37°C for 1 hour. Three μl of cDNA were subjected to PCR using Ready-to-Go PCR beads (Amersham Pharmacia Biotech, Orsay, France) in the presence of Digoxigenin-11-dUTP (Roche, Meylan, France) and 25 ng of Reelin-specific primers [forward primer (5’-AGTGCCTACCTTCCTCTTCCAAT); reverse primer (5’- ATGGGTATCGCCTAAGCGACCTT) (GIBCO BRL, Cergy Pontoise, France)]. Amplification was performed using Genius (Techne, Bibby Sterilin, Nemours, France) thermocycler. The cycling conditions for PCR were 1 cycle of initial denaturation at 95°C for 5 min followed by 40 cycles, each at 95°C for 0.5 min, 63°C for 1 min and then at 72°C for 0.5 min. The expected size of the PCR product (910 bp) was checked by electrophoresis in 1.5% agarose gel.

Digoxigenin labelled probes were tested by the dot-blot method using a nitrocellulose membrane (Millipore, Molsheim, France). The denatured probes were serially diluted and spotted onto nitrocellulose membrane. Spotted probes were then incubated with alkaline phosphatase tagged anti-Dig antibody and detected as described below.

#### *In situ* hybridization

The protocol described in Jalabi et al [19, 20] was adapted to formalin-fixed paraffin sections. Sections were rehydrated and washed twice in SSC2X for 10 min at RT, treated with HCl 0.2 M for 8 min and then rinsed twice in the same buffer for 10 min. They were treated with proteinase K (GIBCO BRL, Cergy Pontoise, France) 50 μg/ml in SSC2X for 30 min at 37°C. Sections were immersed in 0.1M triethanolamine for 5 min, in 0.25% acetic anhydride in 0.1M triethanolamine for 10 min and then washed twice in SSC2X for 5 min. Two μl of diluted probe was added to 100 μl prehybridization solution (50% formamide, 2XSSC, 5% dextran sulfate, 1X Denhardt's and 0.1 mg/ml salmon testis DNA), boiled for 10 min, and snap-cooled on ice.

Sections were incubated in the above hybridization solution, overnight at 43°C. Next day the sections were washed in SSC2X for 30 min, followed by three sequential washes of 30 min in SSC1X, SSC1X and SSC0.5X. After rinsing for 10 min in 0.1M Tris buffered saline, pH 7.5 (TBS), tissue non-specific binding was blocked by incubation of sections in 3% DNA blocking solution (Roche, Meylan, France) for 30 min at RT. After non specific binding blocking, sections were incubated overnight in sheep anti-Dig antibody (Roche, Meylan, France) at 1:500 dilution in TBS followed by 2 hours incubation in biotinylated anti-sheep antibody (Roche, Meylan, France) at 1:200 dilution in TBS. Amplification was performed either with the streptavidin-biotin complex (Vectastain Elite kit, Vector Laboratories, Abcys, Paris, France) or using the catalysed signal amplification system CSA/Per from Dakocytomation according to the instructions of the manufacturer as tyramide amplification system is known to be able to detect very few copies of viral DNA in nuclei [21–23], where other amplification systems do not work. VIP (Vector Laboratories, Abcys, Paris, France) was used as the chromogen. The sections were counterstained with methyl green, air-dried and cover slipped with Eukitt.

Controls for *in situ* hybridization consisted of sections incubated in prehybridization solution without the probe. Other control experiments were performed using sense Dig-labelled probes. In these conditions, no staining was observed.

## Results

### Early development of the olfactory system

**Fig 1:**
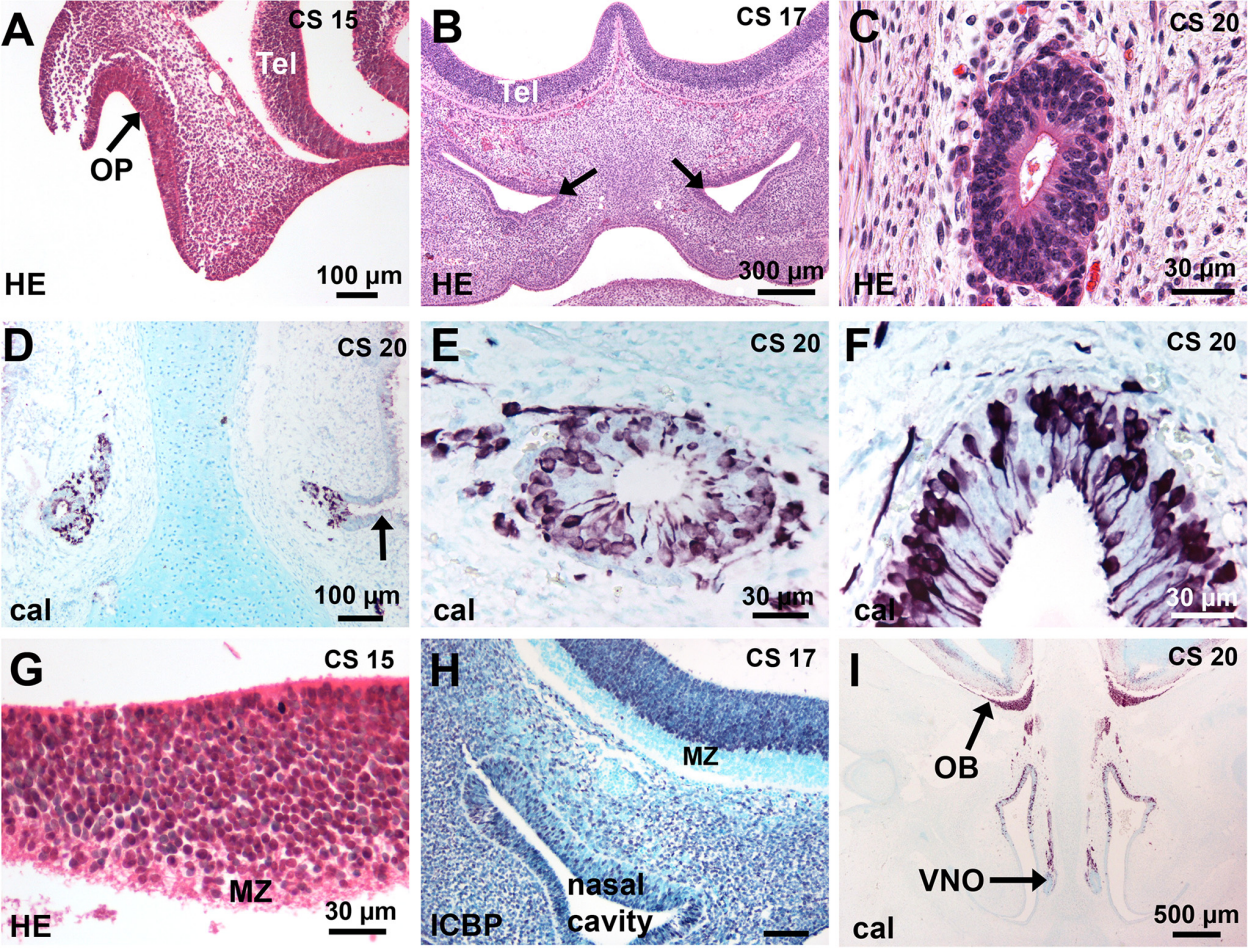
Development of the human olfactory system. (A-F) Development of the OE and VNE. (A) The arrow points to the invaginated OP in front of the telencephalon. (B) The nasal cavities have developed and the presumptive VNO is first delineated (arrows). (C) A transverse section of a VNO shows a small tube lined with a pseudostratified epithelium. (D, E) The entry of the VNO (arrow) is lined with a respiratory epithelium; calretinin-positive cells are present in the VNE and in the cell-stream leaving the VNO. (F) Many calretinin-positive cells are present in the OE. (G-I) Development of the OB. (G) Higher magnification of A shows the basal telencephalon consists of a thick proliferating ventricular zone and a narrow marginal zone. (H) ICBP immunostaining (purple nuclei) shows that the MZ has developed. (I) A low magnification of a coronal section at CS 20 shows that the olfactory fibres have reached the telencephalon and form a distinct layer at the surface of the developing OB; OBs are present with a peripheral fibre layer, a differentiating layer and a ventricular layer. Abbreviations: (cal) calretinin immunostaining, (CS) Carnegie stage, (HE) hematoxylin-eosin staining, (MZ) marginal zone, (OB) olfactory bulb, (OP) olfactory placode, (Tel) telencephalon.

At CS15, the OP was invaginated as a thick epithelium (Fig 1A); at CS16-17, the primordium of the VNO first appeared (Fig 1B) and was well delineated at CS18 in the medial walls of the olfactory pits, protruding towards the mesenchyme. At later stages, VNOs appeared quite identical as small tubes (Fig 1C), communicating with the nasal cavities by a channel lined with a pseudostratified epithelium similar to that of the floor of the nasal cavity (Fig 1D). As previously described, calretinin-positive neurons were present in the VNE as well as in the OE (Fig 1E and 1F).

From CS15 to CS17, the basal telencephalon in front of the OP and OE consisted in a peripheral marginal zone (MZ) as defined by Meyer et al [24] and a ventricular proliferating zone (Fig 1G and 1H); OBs were present at CS 20–21 and easily identified by the strongly calretinin-labeled peripheral olfactory fibre layer (Fig 1I).

### Neuronal migrating cells in the peripheral olfactory system

**Fig 2:**
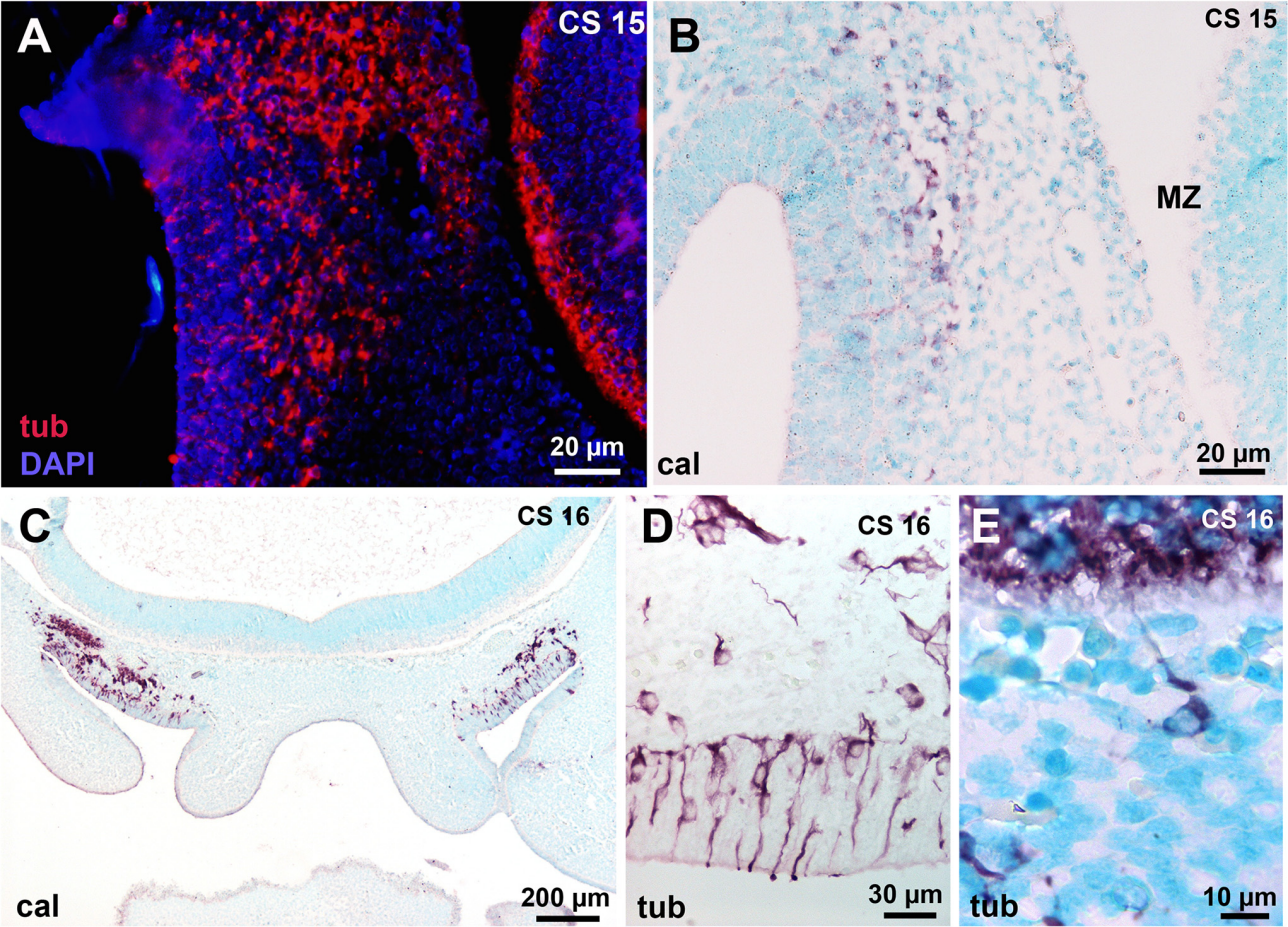
Early neuronal migratory mass in the mesenchyme between the presumptive olfactory epithelium (OE) and the basal telecephalon. (A-B) Same case as in Fig 1A. (A) Beta-III tubulin-positive cells are present in the presumptive OE, the mesenchyme and in the marginal zone of the basal telencephalon. (B) Calretinin immunostaining in the migratory mass and the presumptive OE but not in the marginal zone. (C) Calretinin-positive neurons detach from the OE and form a compact migratory stream in front of the basal telencephalon; no calretinin-positive cells are present in the marginal zone. D: high magnification of the OE; beta-III tubulin-positive neurons are present in the OE and enter the mesenchyme. E: A single beta-III tubulin-positive cell sends a neurite to the telencephalon. Abbreviations: (cal) calretinin immunostaining, (CS) Carnegie stage, (MZ) marginal zone, (tub) beta-III tubuline immunostaining.

At CS 15, beta-III tubulin-positive neurosensorial cells (Fig 2A and 2B) were present in the invaginated OP. This staining was enhanced at later stages (Fig 2C and 2D). Migrating neuronal markers-positive cells, as illustrated by beta-III tubulin and calretinin immunostaining were present in the mesenchyme between the nasal epithelium and the basal telencephalon at CS 15 (Fig 2A and 2B). At C16 enhanced calretinin staining in the mesenchyme shows neuronal cells leaving the epithelium (Fig 2D) and entering the basal telencephalon (Fig 2E). At that time, no olfactory fibres had yet entered the telencephalon. From CS 20–21 to GW14, neuronal cells still detached from the VNE but not from the OE (Fig 1E and 1F).

### Reelin expression in the telencephalon and the peripheral olfactory system

**Fig 3:**
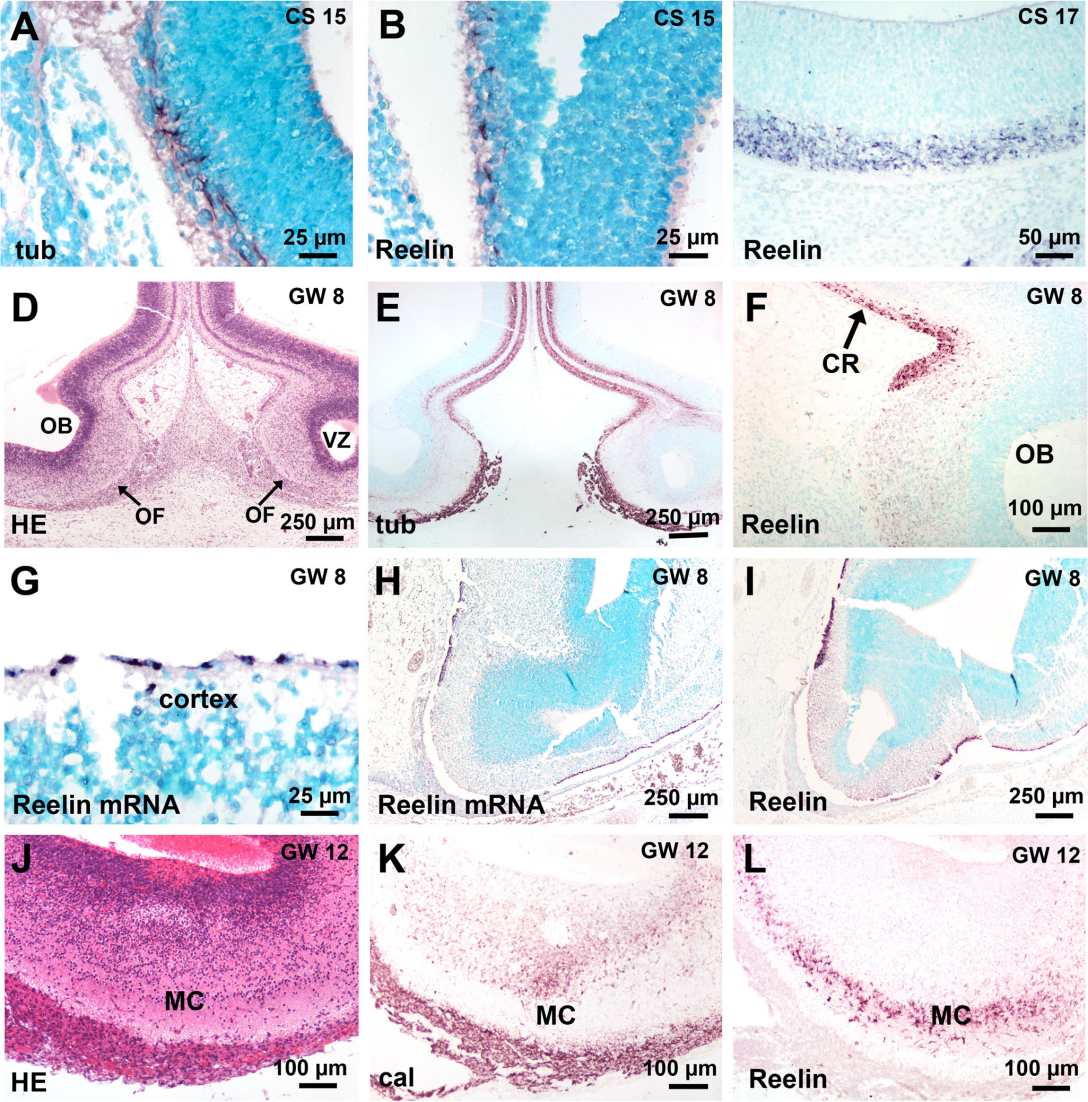
Reelin expression in the telencephalon and in the olfactory bulb (OB). (A, B) Same case as in Fig 1A. Beta-III tubulin- and Reelin-positive cells in the marginal zone of the telencephalon when the olfactory placode has invaginated in front of the telencephalon. (C) A thicker layer of Reelin-positive cells as during the preceding stage is present in the external part of the marginal zone. (D, E) The OB is well developed and shows a thick, beta-III tubulin-positive olfactory fibre layer. (F) The weakly Reelin-positive mitral cells in the OB contrast with the strongly Reelin-positive Cajal Retzius cells. (G) High magnification of the dorsal cortex showing Reelin mRNA in the single layer of Cajal-Retzius cells precursors. (H, I) Nearly adjacent sections comparing Reelin-positive cells by *in situ* hybridization (H) or immunocytochemistry (I). (J-L) At GW 12, the mitral cell layer is well delineated, including calretinin-negative but Reelin-positive cells. Abbreviations: (cal) calretinin immunostaining, (CR) Cajal-Retzius cells, (CS) Carnegie stage, (GW) gestational week, (HE) hematoxylin-eosin staining, (MC) mitral cell layer, (OB) olfactory bulb, (OF) olfactory fibre layer, (tub) beta-III tubuline immunostaining.

At CS15, the narrow external MZ of the telencephalon in front of the invaginated OP contained beta-III tubulin-positive (Figs 2A and 3A), calretinin-negative (Fig 2B) cells aligned tangentially to the pial surface; these neural cells were Reelin-positive (Fig 3B); at CS 16–17, the MZ was thicker and well delineated from the proliferating ventricular zone (Fig 1H); cells in the MZ were beta-III tubulin-positive (Fig 2E), Reelin-positive (Fig 3C) but calretinin-negative (Fig 2C). At CS 20–21, when the OB had developed, the olfactory fibres layer was thick, beta-III tubulin- and calretinin-positive (Fig 1I); at GW8, a clear delineation was present between the weakly Reelin-positive developing mitral cells in the differentiating layer in the OB and the strongly Reelin-positive Cajal Retzius cells as detected by immunocytochemistry or *in situ* hybridization (Fig 3D–3I); at GW12, the mitral layer was well delineated, calretinin-negative and Reelin-positive (Fig 3J–3L).

**Fig 4:**
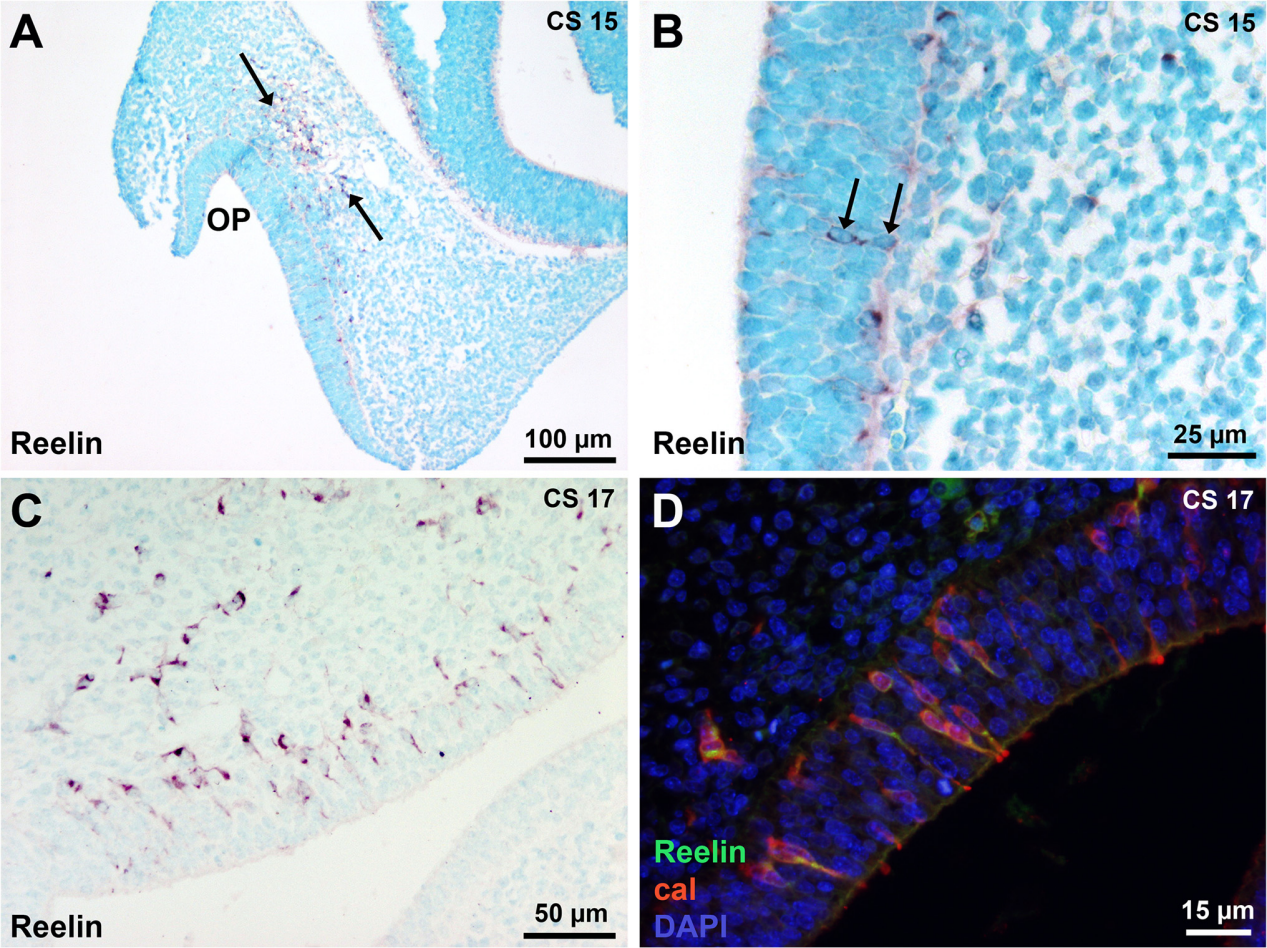
Early Reelin-expressing cells in the peripheral olfactory system. (A) At CS 15, few Reelin-expressing cells are present in the presumptive OE lining the invaginated placode and in the migratory stream in the mesenchyme in front of the telencephalon (arrows). (B) A higher magnification of the epithelium shows two Reelin-positive cells (arrows). (C) At CS 17, more Reeling-positive cells are present in the OE, leaving the OE and entering the migratory stream. (D) Double Reelin and calretinin immunostaining shows that Reelin-positive cells in the OE and the mesenchyme are neurons. Abbreviations: (cal) calretinin immunostaining, (CS) Carnegie stage, (OP) olfactory placode.

A striking observation was the presence of Reelin-expressing cells in the presumptive OE and in the migratory stream between the OE and the basal telencephalon at early developmental stages. At CS15, Reelin-expressing cells were present in the epithelium of the invaginated OP and in the migratory stream (Fig 4A and 4B). At CS 16 and 17, more Reelin-positive cells could be observed in the OE, leaving the OE and entering the migratory stream (Fig 4C). Double Reelin and calretinin immunostaining showed that Reelin-positive cells in the epithelium and mesenchyme from CS15 to CS17 exhibited neuronal identity (Fig 4D).

### Dab 1 expression in the olfactory system

**Fig 5:**
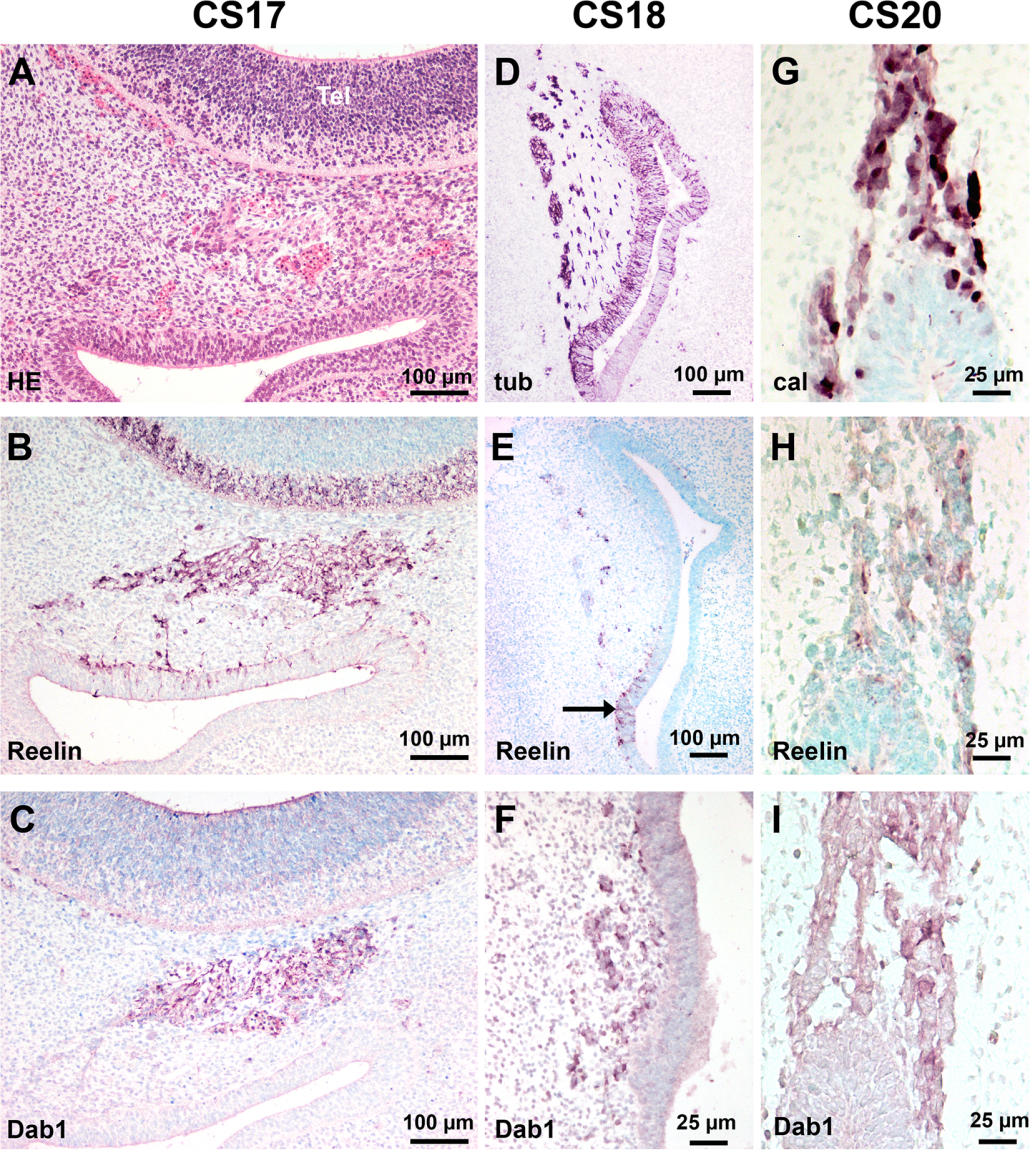
Reelin and Dab 1 expression in the olfactory system. (A-C) Adjacent sections in a CS 17 embryo. (A) The migrating cells in the mesenchyme are well delineated (arrow) in front of the telencephalon which shows a thick proliferating ventricular zone and the developing marginal zone. (B) Reelin is present in the OE, the migrating cells in the mesenchyme and in the marginal zone of the basal telencephalon. (C) Dab 1-expressing cells are present in the migratory mass but not in the OE and the marginal zone. (D-F) Near adjacent sections in a CS 18 embryo. (D) Beta-III tubulin-positive cells are present in the OE, in the presumptive VNE (arrow) and in the cells leaving the presumptive VNE. (E) Reelin is only present in the presumptive VNE and in the cell stream leaving the VNE. (F) Higher magnification of the presumptive VNE: Dab 1 is present in the stream leaving the presumptive VNE but not in the epithelium. (G-I) Adjacent sections at CS 20. (G) Calretinin-positive cells are present in the VNE and in the migrating cell stream leaving the epithelium. (H) Only weakly Reelin-labeled cells are present in the migrating cell stream. (I) Dab 1 is present in the migrating cell stream but not in the VNE. Abbreviations: (cal) calretinine immunostaining, (CS) Carnegie stage.

**Fig 6:**
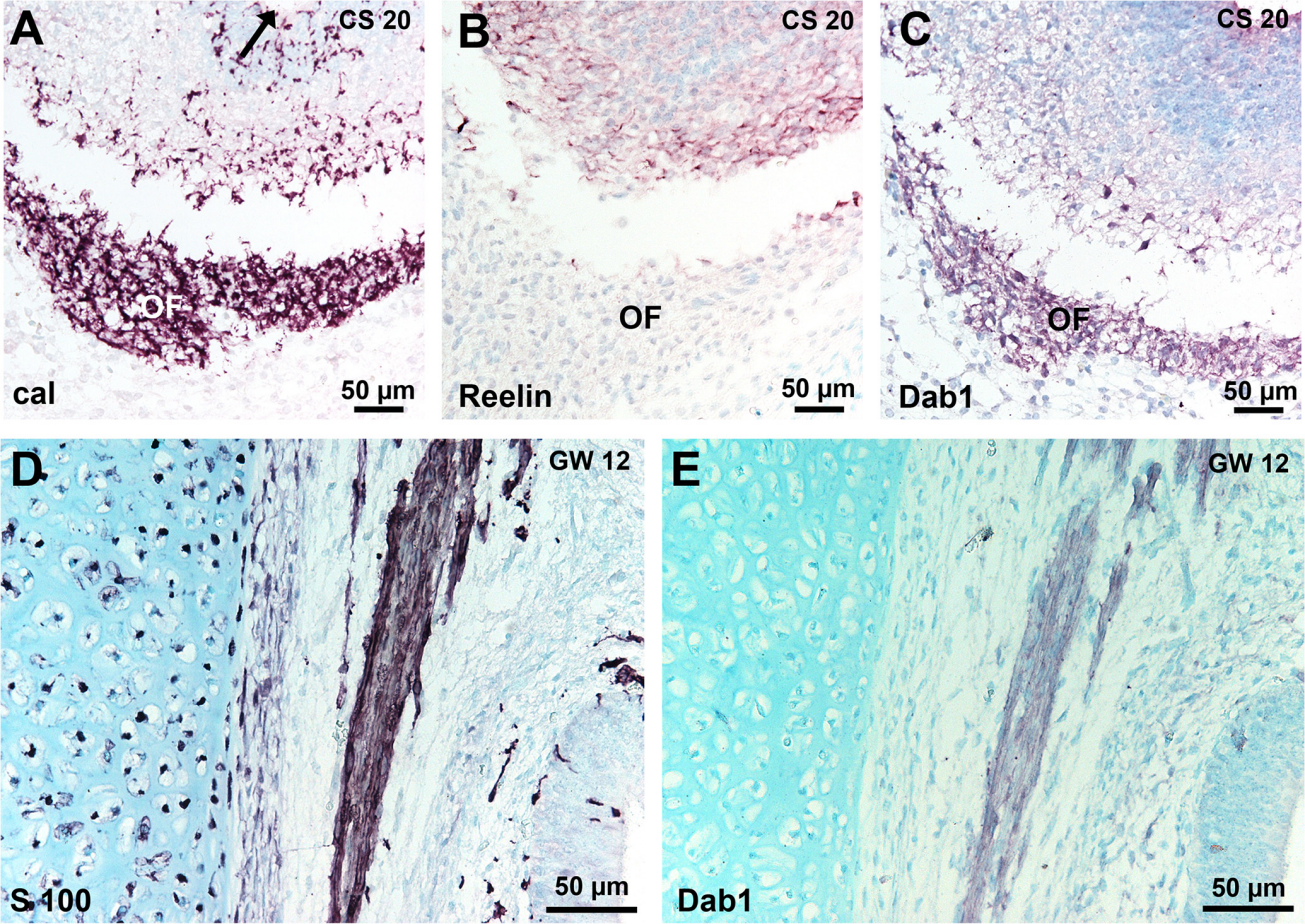
Dab 1 expression in the olfactory nerve. (A-C): Near adjacent sections of the OB at CS 20. (A) Calretinin–positive fibres form a well delineated olfactory fibre layer around the protruding OB after the olfactory fibres have reached the ventricule (arrow). (B) Weak Reelin-staining of the developing mitral cells. (C) Dab 1 expression by cells in the olfactory fibre layer. (D, E) Adjacent sections of the olfactory lamina propria at GW12: Dab 1 and S100 protein expression show a similar pattern in the olfactory fascicles. Abbreviations: (cal) calretinin immunostaining, (CS) Carnegie stage, (GW) gestational week, (OF) olfactory fibre layer.

Since Dab 1 plays a key role in the Reelin signalling pathway, we examined the relation between Reelin and Dab 1 expression. At CS15, when Reelin-positive cells were detected, we did not observe Dab 1 expression in the invaginated OP or in the migrating stream. From CS16 to CS17, both Dab 1 and Reelin were present in the migratory mass of cells in the mesenchyme and no Dab 1 expressing cells were observed in the OE (Figs 4C, 4D and 5A–5C). Double immunostaining of Reelin and Dab 1 did not allow concluding about their colocalisation. At CS 18 and at later stages, no Reelin-positive cells were present in the OE (Fig 5D and 5E). In contrast, the presumptive VNE still contained Reelin-positive cells at CS 18 (Fig 5E), but not at CS 20 (Fig 5H). The migratory flow arising from VNE (Fig 5D and 5G) contained Reelin-positive cells at CS 18 and 20 (Fig 5E and 5H). No Reelin-positive cells were observed in the VN/TN from 7 to 14 GW. From CS 18, Dab 1 was present in the VN/TN and olfactory fascicles, as slender cells around nerve fibres or neurons (Fig 5F and 5I) and in the olfactory fibres layer in the OB (Fig 6A–6C). Dab 1 expression in the olfactory fascicles overlapped that of S100 protein (Fig 6E).

### GnRH neurons were not Reelin-positive

**Fig 7:**
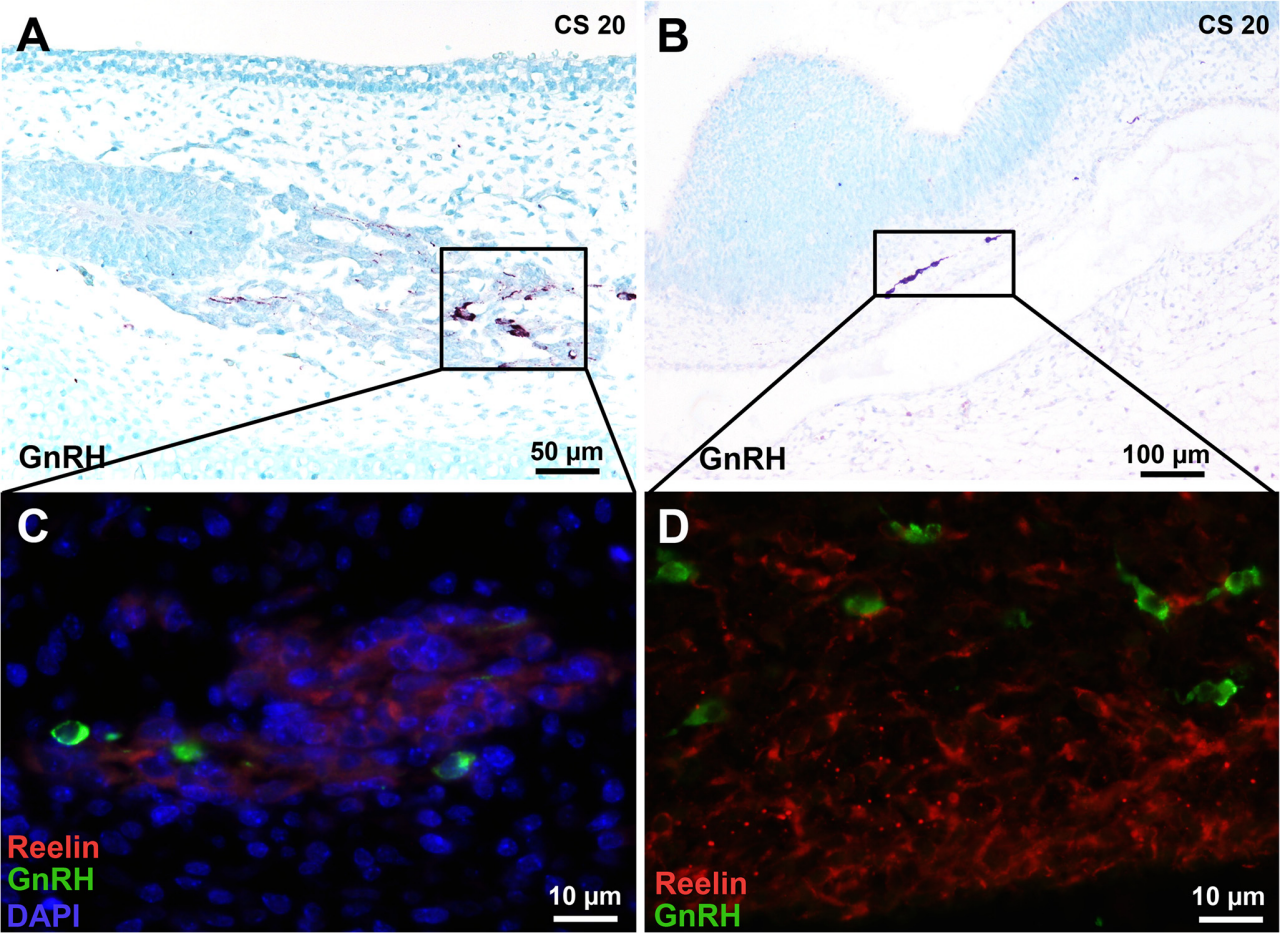
GnRH neurons in the cell stream arising from the vomeronasal organ and in the central nervous system are not Reelin-positive. (A, C) Some cells arising from the VNO are faintly Reelin-positive as in Fig 5H but are distinct from GnRH neurons. (B, D) GnRH cells which have entered the central nervous system are distinct from the Reelin-positive Cajal-Retzius cells present in the marginal zone. Abbreviations: (CS) Carnegie strage.

We first detected GnRH at CS 20 in the migrating stream from the VNO and entering the telencephalon (Fig 7A and 7C); double staining showed that Reelin- and GnRH-expressing cells were distinct populations both in the telencephalon and in the VN/TN fascicles (Fig 7B and 7D).

## Discussion

We show that during early olfactory development in human, neuronal cells arising from the presumptive OE/VNE express Reelin; this is a temporally and spatially regulated phenomenon since cells first arising from the presumptive OE and then from the presumptive VNE were Reelin-positive. This expression in the epithelium and migrating stream was a transient phenomenon since no neuronal Reelin-positive cells were present from GW7 to GW14.

Olfactory area development for each stage studied was in accordance with previous studies [25–27]. Bystron et al [28] observed beta-III tubulin-positive neurons in the OP and in the adjacent mesenchyme as soon as CS13. We showed at CS15, when the OP has invaginated, the presence of neurons both in the epithelium and the adjacent mesenchyme. The neurons arising from the OP, so called pioneer neurons [29] were seen penetrating the telencephalon at CS15, as we did at CS16. A common origin of these pioneer neurons with early cortical predecessor neurons in the anterior neural plate has been suggested [28]; the same authors concluded that these cells were not Cajal-Retzius cells as they did not detect Reelin at that time. However, we detected Reelin-expressing neurons in the basal telencephalon, as well as in the epithelium and in the mesenchyme at CS15. A point we could not elucidate was whether the neurons entering the telencephalon were Reelin-positive; if that was the case, we could hypothesize that the very early Reelin-positive cells at the surface of the presumptive cortex could be in part of peripheral origin. That point needs further explorations using both immunocytochemistry and *in situ* hybridization. Indeed, we successfully used for Reelin mRNA detection, an *in situ* hybridization method combining probe preparation by PCR, as previously described by Jalabi et al [19, 20] for oligodendrocytes PLP mRNA detection, and the powerfull signal amplification by tyramide, we previously used to detect single viral copies [21–23]. While classical streptavidin-biotin complex method gave no signal, tyramide amplification showed strong Reelin signals. One constraint of *in situ* hybridization is the tissue fixation method since Bouin-Holland fixative preserves morphological details, but destroys nucleic acids, reason why we could not apply the method to the youngest embryos in the present study. This powerfull signal amplification method by tyramide will be useful when working on human embryonic and foetal tissue paraffin-embedded sections, mainly in pathological conditions, where mRNA levels may be very weak because of the delay between *in utero* death and tissue fixation.

The most studied neuronal population in the migratory mass [30] are GnRH neurons that migrate in human as in other species from the OP to the hypothalamus [2, 31, 32]; they have been first detected at 42 but not at 28 or 32 days of embryogenesis [31]. Our observations highly suggest that the early neurons expressing Reelin were not GnRH neurons. The first Reelin-positive neurons in this study, present at CS 15 (approximately 35 days of pregnancy) were GnRH-negative. At CS20, when GnRH neurons very strongly expressed the peptide, the very few Reelin-positive cells still present in the VN/TN were not GnRH neurons.

What may be the role of the early Reelin-positive neurons? In the central nervous system, Reelin is an extracellular glycoprotein essential for migration and correct positioning of cerebral cortical neurons as well as for other neurons such as midbrain or spinal cord neurons. For the peripheral olfactory system, it has been suggested in mice that olfactory and vomeronasal fibres pathfinding was dependent upon the presence of Reelin in the mesenchyme [13]; however, in *reeler* mice where Reelin is absent, normal VN development occurs [12] suggesting that Reelin is not the only guidance molecule for olfactory or vomeronasal fibres. However, it has been shown that Schwann cells in the developing peripheral nerve secrete Reelin, its level is downregulated in adult but upregulated following nerve injury and its abscence in *reeler* mouse impairs axonal regeneration following injury [33, 34]. In the present study in human, we only observed Reelin-expressing neurons in a temporally determined window, first in the presumptive OE and then in the presumptive VNE; the expression is correlated with the early detachment of cells from the epithelium since soon after the beginning of epithelium differentiation, the intra-epithelial expression ceased. As Reelin is mainly considered as acting as a secreted extracellular molecule binding to receptors at the cell surface of other cells as those which secrete Reelin, we studied the expression of Dab 1, an adaptator intracellular protein activated following Reelin binding to its receptors (reviews in [35–38]). We did not detect Dab 1, neither in the epithelium nor in the mesenchyme at the earliest stage when epithelial and intra-mesenchymal neurons were Reelin-positive. The question arises whether the expression level of Dab 1 was too weak at that time to be detected by our immunocytochemistry method. Further studies are needed to explore the expression of the other members of the Reelin-pathway to elucidate that point. Conversely, Reelin may act independently from Dab 1; one possibility may be an autocrine action. Indeed, Reelin expressed by lymphatic endothelial cells during development in rodents and human [11] up-regulates an endothelial factor promoting smooth muscle development leading to lymphatics morphogenesis, in a way independent from the classical Reelin receptors [39]. Also Cariboni et al [40] showed that Reelin was a repellent for GnRH, guiding the neurons to the hypothalamus, in a manner independent from the classical Reelin cascade. Whether pioneer neurons promote their own migration through Reelin expression and action in an independent manner from the classical Reelin cascade will need further consideration.

At CS18, Dab 1 was present around and within olfactory fascicles, when olfactory Reelin expression had ceased; the cells shared the same pattern as S100 protein-positive cells, suggesting they are OECs. OECs are considered as a distinct glial type showing common properties of both Schwann cells and astrocytes. They promote the outgrowth of newly formed olfactory axons and direct them to their proper position in the OB (for review see [41, 42]). As Reelin was no more present, Dab 1 may function in a Reelin-independent pathway. Andrade et al [43] showed that postnatal neuronal migration in the rostral stream needs ApoER2/VLDL receptors and Dab 1, independently of Reelin. However, Howell et al [44] showed that VEGF regulated NMDA receptor via Dab 1. Dab 1 present in the human vomeronasal cell clusters or in the olfactory nerve fascicles may then mediate other signaling cascade as that initiated by Reelin.
